# Correction to “Treating Hematological Malignancies With OR‐2100, an Orally Bioavailable Prodrug of Decitabine”

**DOI:** 10.1111/cas.70230

**Published:** 2025-10-20

Watanabe, T., Kidoguchi, K. and Kimura, S. (2025), Treating Hematological Malignancies With OR‐2100, an Orally Bioavailable Prodrug of Decitabine. *Cancer Sci*, 116: 853–861. https://doi.org/10.1111/cas.16452

In Table 1, the reference citations were incorrect. The correct table is shown below:

**TABLE 1:** Efficacy and molecular mechanisms of OR‐2100 in mouse models of hematological malignancies.

Disease	Treatment	Molecular mechanisms (key molecules)	References
Colon cancer	Monotherapy		[14]
MDS/AML	Monotherapy	Induction of differentiation‐related genes	[28]
ATL	Monotherapy	Induction of negative regulators of TCR signaling	[30]
Lymphocytic leukemia	Monotherapy	Down‐regulation of MYC expression	[48]
MDS/AML	Combination therapy (venetoclax)	Induction of ROS, down‐regulation of VAMP7	[18]
CML	Combination therapy (ABL TKI)	Induction of PTPN6	[55]
ALCL	Combination therapy (ALK TKI)	Down‐regulation of Wnt/β‐catenin pathway	[62]
ATL	Combination therapy (EZH inhibitor)	Induction of DUSP5	[21]

Figure 3 legend was incorrect. The correct legend is shown below:

**FIGURE 3** OR‐2100 prevents leukemogenesis associated with aberrant DNA methylation in AKR mice. Tumor cells isolated from AKR mice have hypermethylated proximal promoter regions compared with normal T cells (left panel). Kaplan–Meier survival curves of AKR mice treated with vehicle, OR‐2100, or DAC (right panel) [48]. Part of material from: Yuta Yamamoto et al., DNA demethylating agents for chemoprevention of oncovirus‐associated leukemogenesis, *Leukemia*, 2024, Springer Nature.

In the third paragraph of the section “2.3 OR21 Monotherapy in ATL,” the text “Several genes that negatively regulate T‐cell receptor signaling, including *THEMIS*, *LAIR1*, and *RNF130*, were down‐regulated through promoter hypermethylation in ATL cells [16].” was incorrect.

This should have read: “Several genes that negatively regulate T‐cell receptor signaling, including *THEMIS*, *LAIR1*, and *RNF130*, were down‐regulated through promoter hypermethylation in ATL cells [30].”

We apologize for these errors.

